# Incidental detection of *Candida auris* in an orthopedic patient at a Danish university level-II trauma center

**DOI:** 10.2340/17453674.2025.44954

**Published:** 2025-12-11

**Authors:** Misk G F MUHAMMAD, Christian BERG, Henrik CALUM, Poul PEDERSEN, Emilia FUSARU, Sofie K HØJ, Helene B GYRUP, Mette PINHOLT, Tazio MALEITZKE, Mette D BARTELS

**Affiliations:** 1 Trauma Orthopaedic Research Copenhagen Hvidovre (TORCH), Department of Orthopaedic Surgery, Copenhagen University Hospital – Amager and Hvidovre, Hvidovre; 2 Department of Clinical Microbiology, Copenhagen University Hospital – Amager and Hvidovre, Hvidovre; 3 Department of Clinical Medicine, University of Copenhagen, Copenhagen, Denmark; 4 Charité – Universitätsmedizin Berlin, corporate member of Freie Universität Berlin and Humboldt-Universität zu Berlin, Center for Musculoskeletal Surgery, Berlin; 5 Berlin Institute of Health at Charité – Universitätsmedizin Berlin, Julius Wolff Institute, Berlin, Germany

This case report describes the handling and consequences of the incidental finding of *Candida auris*, a yeast that has become a major global health concern due to its ability to persistently colonize patients and to develop multiresistance [1]. *C. auris* was found in a urine sample of an orthopedic patient at a level II trauma center in Denmark. A 68-year-old Danish man, who has been living in Turkey for the last 17 years, arrived at the emergency department of a regional hospital with necrotic foot ulcers. He was transferred to the emegency depoartment of our level II center. Medical history revealed poorly managed type 2 diabetes mellitus and 2 previous strokes, resulting in partial paralysis and expressive aphasia. The patient had suffered from chronic diabetic foot ulcers on both feet for approximately 1.5 months, with signs of local infection but without systemic symptoms. The patient was wheelchair bound. In Turkey, the patient was treated at home by a nurse for his ulcers.

Upon initial examination, the patient was alert, afebrile, and hemodynamically stable (BP 168/80 mmHg, heart rate 102 bpm). The right foot showed 4 ulcers with exposed tendons, purulent discharge, reduced sensation, weak peripheral pulses, and delayed capillary response. The left foot had one superficial ulcer and intact pulses. Laboratory parameters showed hyperglycemia (40 mmol/L), elevated CRP (58 mg/L), hyponatremia (124 mmol/L), metabolic acidosis, anemia (Hb 5.3 mmol/L), and kidney failure (eGFR 20). Blood and urine cultures were obtained. Intravenous piperacillin-tazobactam, insulin, fluids, and blood transfusions were initiated. Imaging and vascular assessments showed only moderate healing potential (ankle: 117 mmHg; toe: 43 mmHg). In agreement with the patient, a transfemoral amputation was performed the following day.

Key action points to consider

- **Immediate isolation and coordinated response**
  As soon as *Candida auris* is identified, isolate the patient and initiate close collaboration between infection control, clinical microbiology, and ward staff to prevent spread.
- **Comprehensive screening and targeted cleaning**
  Perform multi-site screening of the index and exposed patients, and implement intensified environmental cleaning with chlorine and hydrogen peroxide vapor disinfection.
- **Rapid communication and risk-based decision-making**
  Ensure prompt notification of all relevant departments and evaluate the need for ward closure or cohorting based on transmission risk, to balance infection control with resource use.

The urine sample taken on admission showed growth of 10^5^/mL *Escherichia coli,* 10^5^/mL *Enterococcus faecalis* and 10^3^/mL yeast. In accordance with the urine culture laboratory instruction, a low amount of yeast in a polymicrobial culture is not further investigated. A catheter urine sample was taken 3 days after admission and 2 days later a monoculture of 10^5^/mL *C. auris* was identified using MALDI-TOF mass spectrometry (Bruker; https://www.bruker.com/en.html). As the patient showed no signs of a urinary tract infection, the finding was considered a colonization, and no antifungal treatment was initiated. The urine sample from the admission date was re-analyzed and *C. auris* was identified, with the conclusion that the patient was already colonized with *C. auris* on admission. The orthopedic consultant on call was promptly notified by a clinical microbiologist when *C. auris* was identified and the infection control unit was immediately involved.

The index patient was isolated with contact precautions. The patient was screened for nose, axillae, groin, wound, and rectum *C. auris* colonization. His 2 fellow-patients were isolated in a separate room and screened for *C. auris*. As the index patient had not been in isolation the first 5 days of his hospital stay, there was an increased risk of spread to patients outside of his room, and therefore a decision to temporarily close the orthopedic ward to new admissions was made. Patients already admitted were screened for *C. auris* and were only permitted to be discharged prior to a negative culture result if they could be discharged directly to their own home and did not require healthcare services at home. These precautions were taken to avoid the spread of *C. auris* to the primary care sector. Cleaning of the department was intensified, e.g., daily chlorine cleaning of all contact points. Additionally, the operating theater in which the patient was operated on was temporarily closed and disinfected with hydrogen peroxide vapor [2]. The operating theater was re-opened the following day with standard cleaning procedures. 4 patients who had been operated on in the same operating theater after the index patient were placed in isolation as a precautionary measure and screened for *C. auris*. Healthcare staff were not included in the screening process, in line with recommendations from the CDC [3]. *C. auris* was found in the groin of the index patient, but no other patients were found to be colonized with *C. auris* and the orthopedic ward was reopened for admissions after 3 days. The index patient stayed in the hospital for 14 days, and during his stay, weekly screenings for *C. auris* were performed for all patients who had been hospitalized in the orthopedic ward for 7 or more days according to regional guidelines [4]. The screening procedure was continued for 2 weeks after the discharge of the index patient and *C. auris* was not found in any other patients or the environment following post-discharge environmental sampling. After discharge from the hospital, the patient was transferred to a rehabilitation unit in another region of Denmark.

## Ethics and disclosures

Oral and written informed consent for publication was obtained from the patient. Complete disclosure of interest forms according to ICMJE are available on the article page, doi: 10.2340/17453674.2025.44954

## Discussion

*C. auris* was first identified in 2009 in pus from the ear of a Japanese patient [1]. *C. auris* has become a major global health concern due to its ability to persistently colonize patients and survive in healthcare environments for prolonged periods which necessitates enhanced cleaning and stringent infection prevention protocols [5,6]. High mortality rates are reported for invasive infections [7]. More than 90% of *C. auris* strains are resistant to fluconazole [8] and some strains have evolved a pan-resistance, rendering all available treatments ineffective [7].

Outbreaks of *C. auris* require many healthcare resources and coordinated efforts to facilitate ward closures, and redirection of patients [9]. To address these challenges, close collaboration between the infection control unit, involved clinical departments, the support services department, and hospital management is crucial to control a possible outbreak.

In this case report we have outlined how a Danish level II trauma center handled the finding of *C. auris* in a surgical healthcare setting. As this was the first finding of *C. auris* in the hospital and only very few cases had previously been identified in Denmark, we based our risk assessment on the literature, where *C. auris* is described as a pathogen with a very high potential for transmission and risk of causing larger outbreaks in healthcare settings [10]. As the patient had not been isolated during the first 5 days of his hospital stay, we assumed that the transmission risk was increased, which led to ward closure until screening results of all admitted orthopedic patients were available [11,12]. The ward remained closed for 3 days, due to the requirement of 48 hours of culturing for *C. auris*. A recent study by Jolivet et al. on qPCR testing for *C. auris* found that in addition to being much faster, qPCR was also more sensitive than culturing [13]. No PCR test was available in the department of clinical microbiology because of the rare occurrence of *C. auris* in Denmark but should be considered in the future.

To limit cross-contamination and ensure containment, temporary closures of affected units, deep cleaning, and gradual reopening with strict environmental monitoring have been shown to be effective in outbreak scenarios [12]. However, the cost of temporarily closing a ward must be weighed against the risk of an outbreak. In the case described, we saw no spread to other patients even though the index patient had not been isolated for the first 5 days of his admission. Thus, we presumed that the general infection control measures in the hospital setting were effective, and in a similar situation in the future we would probably not close an entire ward until we know whether *C. auris* has spread to fellow-patients.

According to Danish national guidelines [9], patients who have been hospitalized outside the Nordic European countries within the last 6 months are considered at risk for *C. auris* colonization. It is recommended that these patients be screened for multidrug-resistant bacteria and *C. auris* at the time of admission. The index patient did not fulfil this criterion for screening on admission; however, the patient was treated at home by a nurse for his foot ulcers. Following this case, we are considering whether this criterion should be added to the screening algorithm.

Precautionary measures that were taken at our hospital, including immediate patient isolation with contact restrictions, multi-site screening of the index patient and exposed patients, intensified chlorine cleaning, and hydrogen-peroxide vapor (HPV) decontamination, were in agreement with reports from an intensive care unit (ICU) in the UK, a hemodialysis center in New Jersey, NC, US, as well as consensus guidelines from the CDC and an expert meeting of infection prevention and mycology experts [12,14–16]. In accordance with our approach, multi-site screening including axilla and groin swabs is generally recommended when screening for *C. auris* [3,17,18]. Immediate single-room isolation and supply of staff with personal protection equipment (PPE) is also in line with previously published reports and guidelines [12,14,16]. We used daily chlorine cleaning and targeted HPV for the operating theater and the patient’s room following discharge. This aligns with in-vitro studies and expert guidance showing that *C. auris* is less susceptible to quaternary ammonium compounds compared with chlorine (≥ 1,000 ppm), hydrogen peroxide (including vapor), and peracetic-acid formulations, where germicidal efficacy depends on formulation and contact time [2,14,19,20].

We temporarily closed the orthopedic ward to new admissions pending culture results. In contrast, previous reports from ICUs and hemodialysis units have emphasized the establishments of dedicated cohort areas, implementation of traffic control, and adaptation of patient flows—for example through dedicated or terminally disinfected machines and adjusted seating or scheduling [15,16]. Our decision to temporarily close the ward required careful consideration and involvement of hospital management and will always depend on the local situation. Overall, our response aligned with those described for other high-risk settings regarding isolation, contact precautions, screening, and disinfection.

Patient transfer to other healthcare facilities involves careful planning when a patient is colonized with *C. auris* [12] and therefore our infection control nurses notified the rehabilitation unit well in advance of the transfer. *C. auris* colonization can persist for months to over a year with a median persistence of approximately 2–3 months. However, prolonged persistence beyond a year is common, particularly in patients with risk factors that include critical illness, implants, or recent antimicrobial treatment [14,21,22]. Clearance is typically defined as 2 to 3 consecutive negative screening results, but relapses may occur [12,14], and the sensitivity of a single screening result is limited [8,18]. Accordingly, we decided to screen the fellow-patients who were most at risk for *C. auris* twice before terminating the isolation.

Overview of key actions due to the finding of *C. auris*

Key actions and details

1. *Isolation precautions*
  Day 0 of intervention: Contact precautions for index patient, 2 fellow-patients who had shared a room with the index patient, and 4 patients who had been in the operating theater after the index patient before it was closed.
2. *Screening for C. auris*
  Day 0 of intervention: Index patient, fellow-patients and all patients in the orthopedic ward (n = 30) plus in adjacent orthopedic wards (n = 20), where 2 of the patients, who had been in the operating theater following the index patient, were admitted, were screened (nose, axillae, groin, rectum plus urine if catheterized and wounds if any). Isolated fellow-patients were screened twice before isolation was terminated on day 6 of intervention. Weekly screenings of patients who had been on the ward for a minimum of 7 days were performed until 2 weeks after discharge of the index patient (until day 23).
3. *Ward closure and cleaning protocol*
  The ward of the index patient was closed on day 0. The index patient had 2 rooms; the primary room was cleaned daily including chlorine disinfection and every third day the patient was moved to the secondary room while hydrogen peroxide vapor was used in the primary room. Rooms of isolated fellow-patients were cleaned daily with chlorine disinfection until day 6 when their isolation was terminated. Daily chlorine disinfection was performed on contact points of the ward until the index patient was discharged (from day 0 to day 10).
4. *Operating theater closure and cleaning protocol*
  The operating theater was disinfected with chlorine and hydrogen peroxide vapor on day 0.
5. *Handling of other areas where the index patient had been*
  The emergency room at 3 hospitals, the post-anesthesia care unit (PACU), and the department of radiology were informed on day 0 of intervention. The rooms where the index patient had been were disinfected with hydrogen peroxide vapor and contact points on the PACU were disinfected with chlorine on day 0.
6. *Reopening of ward*
  The ward was reopened when negative screening results from all patients were available (day 3).

### Conclusion

*C. auris* detection requires quick and coordinated actions including microbiological testing, screening, patient isolation, intensified cleaning protocols, and close collaboration between clinical staff and the infection control unit to reduce the risk of an outbreak. In the presented case, *C. auris* did not spread to fellow-patients, even though the indiex patient had not been isolated for the first 5 days after admission indicating that the general infection control procedures were effective. In future, we will likely refrain from closing an entire ward in a similar situation, but await the screening results of fellow-patients, especially if fast detection by PCR has been implemented. We consider, however, extension of the current screening criteria and suggest some key action points (Table).

## Notes

MGFM: Drafted the manuscript, coordinated revisions, and critically revised the manuscript. CB: Contributed to microbiological analysis and manuscript revision. HC: Provided microbiological expertise and contributed to manuscript revision. EF, HBG, MP, PP, SKH: Assisted in clinical management and manuscript revision. TM, MDB: Supervised the project, contributed to study design, and critically revised the final manuscript.

Handling co-editor: Marianne Westberg

*Acta* thanks Staffan Tevell and Danguole Vaznaisiene for help with peer review of this manuscript.
